# Trypanosomal mitochondrial intermediate peptidase does not behave as a classical mitochondrial processing peptidase

**DOI:** 10.1371/journal.pone.0196474

**Published:** 2018-04-26

**Authors:** Priscila Peña-Diaz, Jan Mach, Eva Kriegová, Pavel Poliak, Jan Tachezy, Julius Lukeš

**Affiliations:** 1 Institute of Parasitology, Biology Centre, Czech Academy of Sciences, České Budějovice (Budweis), Czech Republic; 2 Faculty of Science, Charles University, Prague, Czech Republic; 3 Faculty of Science, University of South Bohemia, České Budějovice (Budweis), Czech Republic; Louisiana State University, UNITED STATES

## Abstract

Upon their translocation into the mitochondrial matrix, the N-terminal pre-sequence of nuclear-encoded proteins undergoes cleavage by mitochondrial processing peptidases. Some proteins require more than a single processing step, which involves several peptidases. Down-regulation of the putative *Trypanosoma brucei* mitochondrial intermediate peptidase (MIP) homolog by RNAi renders the cells unable to grow after 48 hours of induction. Ablation of MIP results in the accumulation of the precursor of the trypanosomatid-specific trCOIV protein, the largest nuclear-encoded subunit of the cytochrome *c* oxidase complex in this flagellate. However, the trCOIV precursor of the same size accumulates also in trypanosomes in which either alpha or beta subunits of the mitochondrial processing peptidase (MPP) have been depleted. Using a chimeric protein that consists of the N-terminal sequence of a putative subunit of respiratory complex I fused to a yellow fluorescent protein, we assessed the accumulation of the precursor protein in trypanosomes, in which RNAi was induced against the alpha or beta subunits of MPP or MIP. The observed accumulation of precursors indicates MIP depletion affects the activity of the cannonical MPP, or at least one of its subunits.

## Introduction

A wide majority of proteins constituting a typical mitochondrion is nuclear-encoded and translocated into the organelle via a dedicated translocation machinery (for reviews see [1–5]). The import of a matrix-targeted protein is initiated by the recognition of its mitochondrial targeting signal located mostly at its N-terminus [6,7]. It is bound by the translocase of the outer membrane (TOM) complex, particularly the TOM20 and TOM22 receptors, and guided through the channel formed by TOM40 (for review see [8]). Next, the pre-protein engages in an interaction with the translocase of the inner membrane (TIM). The TIM machinery facilitates import of the pre-protein through the inner membrane and with the assistance of import motor PAM, the pre-protein is translocated into the organellar matrix (for reviews see [5,9,10]).

Once in the mitochondrial matrix, the pre-protein has its N-terminal targeting signal cleaved off by the mitochondrial processing peptidase (MPP) [11–13]. MPP is a heterodimer formed by the alpha-MPP recognition subunit and the beta-MPP subunit with a catalytic activity [14–16]. A small group of mitochondrial proteins requires a second cleavage after MPP processing, a step that is catalyzed by either the inner membrane peptidase (IMP) [17], the intermediate cleaving peptidase 55 (Icp55) [18] or the mitochondrial intermediate peptidase (MIP) [19]. Together with thimet peptidase, the latter enzyme belongs to the processing peptidases from the M3 family of proteases [20].

MIP was first isolated from rat liver and characterized as a monomeric 75 kDa metallopeptidase [21,22]. Following the R-2 cleavage by MPP within the motif (R)X | (F/L/I)XX(T/S/G)XXXX | [23], a bulky hydrophobic residue (F/L/I) becomes exposed at the N-terminus of the peptide for MIP recognition, which typically cleaves off 8 amino acids [22,24]. The cleavage of this “octapeptide”gave origin to the name of the protein, Oct1, in the yeast *Saccharomyces cerevisiae* [25]. A mutation in the active site of Oct1 rendered the yeast unable to grow solely on non-fermentable carbon source, implying the protein was involved in the biogenesis of subunits of respiratory complexes [26]. The phenotype contrasted with that of mutants in alpha- and beta- subunits of MPP, which was lethal regardless of growth conditions [27]. It was shown recently that *Arabidopsis thaliana* MIP does not display the same activity as its counterparts in yeast and rat mitochondria, since it does not require MPP to trim the N-terminus of the imported proteins prior to its action [28]. While it has been shown that the *A*. *thaliana* MIP differs from its homologs in other eukaryotes by not cleaving off an octapeptide, its mechanism of action remains largely unknown [28].

*Trypanosoma brucei* is a parasitic protist responsible for African trypanosomiasis and belongs to the eukaryotic supergroup Excavata [29]. Recently, a study of *T*. *brucei* outer mitochondrial membrane translocases revealed their distinct composition from other eukaryotes [30]. Nevertheless, this model trypanosome possesses a canonical MPP that has been characterized. Down-regulation by RNAi of the alpha or beta subunits of the MPP heterodimer was lethal for the procyclic stage of *T*. *brucei* [31,32]. No other processing peptidase has been characterized in trypanosomes apart from the above-mentioned canonical MPP, hence our interest in addressing the mechanisms behind the homologue of the MIP.

In this report, we fused the N-terminus of a putative substrate of MIP to yellow fluorescent protein (YFP), and the construct was subsequently transformed and expressed in the inducible RNAi cell lines for *T*. *brucei* MIP, alpha-MPP and beta-MPP, producing a mitochondrially-localized YFP fusion. Upon RNAi induction of MIP, the YFP chimera failed to be processed and accumulated in the same fashion as it did when alpha-MPP and beta-MPP were down-regulated. These results indicate that in *T*. *brucei* MIP does not cleave an octapeptide, and its downregulation affects the expression and activity of at least one of the subunits of the cannonical MPP. The mechanism behind this effect, however, remains to be elucidated.

## Materials and methods

### Bioinformatic analysis of *T*. *brucei* MIP and putative mitochondrial pre-sequences

The gene encoding a putative MIP was identified using BLAST in the *T*. *brucei* genome database (www.tritrypdb.org). The *T*. *brucei* MIP sequence together with 32 selected MIP homologs, 7 genes of closely related thimet peptidases, 9 genes of OpdA and 6 genes of Dcp (S1 Table) obtained by a BLAST search were aligned using Muscle 3.8.425 software (default parameters) [33] and trimmed with BMGE 1.12 (-b 5 -m BLOSUM30) [34]. Protein evolution model was selected by ProtTest 3.2 [35] to reconstruct phylogenetic tree by PhyML 3.1 (topology search: best of NNIs and SPRs, initial tree: BioNJ, Substitution model: LG, proportion of invariable sites: fixed (0), gamma distribution parameter: estimated; number of categories: 4; bootstrap replicates: 500) [36] and MrBayes 3.2.6 (rate matrix: LG; rate variation: gamma; gamma categories: 4; chain length: 2,000,000; heated chains: 4; heated chain temp: 0.2; burn-in length: 500,000) [37].

### Testing of putative MIP substrates

Putative mitochondrial pre-sequences and the cleavage sites of MPP were predicted by Gavel’s consensus patterns search in the PSORTII program (http://psort.hgc.jp/form2.html) [38,39] and MitoProt II [40], using the *T*. *brucei* mitoproteome dataset [41]. Proteins with predicted pre-sequences were further analyzed for the MIP cleavage motif (F/L/I)XX(T/S/G)XXXX [42] within the octapeptide which immediately follows the MPP cleavage site. From the proteins found by this procedure, one was selected to be further analyzed for MPP and MIP proteolysis detection.

### Preparation of cell lines

The procyclic stage of *T*. *brucei* strain 29–13 [43] was used as parental cell line for all experiments. A summary of all cell lines prepared for the purpose of this study is available in Table 1. Cultivation of procyclic *T*. *brucei* was done in SDM-79 media with the respective drug selection. Cell lines in which alpha-MPP (Tb927.2.4110) and beta-MPP (Tb927.9.4520) can be inducibly silenced by RNAi were described elsewhere [31]. MIP RNAi cell line (Tb927.10.9820) was prepared using a pLew100-based stem-loop construct.

**Table 1:** Cell lines constructed for the characterization of *T*. *brucei* MIP. Cell lines used in this study are based on the parental 29–13 procyclic stage (WT), which stably expresses T7 polymerase and tetracycline repressor, under the resistance cassettes of hygromycin and G418, respectively. Inducible stem-loop RNAi and constitutive expression constructs were targeted to rRNA loci for insertion by homologous recombination. ROI = region of interest.

Cell line name	Parental	ROI	Stably transfected construct	Type	Drug resistance
MIP RNAi	29–13	MIP ORF	Stem-loop RNAi	Stem-loop RNAi	Blasticidin
		Beta-MPP-V5	Beta-MPP-V5	*In situ* tagging	Puromycin
Alpha-MPP RNAi	29–13	Alpha-MPP ORF	Stem-loop RNAi	Stem-loop RNAi	Blasticidin
		Beta-MPP-V5	Beta-MPP-V5 cassette	*In situ* tagging	Puromycin
Beta-MPP RNAi	29–13	Beta-MPP ORF	Stem-loop RNAi	Stem-loop RNAi	Blasticidin
		Beta-MPP-V5	Beta-MPP-V5	*In situ* tagging	Puromycin
NDH-YFP Oct	MIP RNAi	NDH+YFP	pABPURO	Constitutive expression	Puromycin
NDH-YFP alpha	Alpha-MPP RNAi	NDH+YFP	pABPURO	Constitutive expression	Puromycin
NDH-YFP beta	Beta-MPP RNAi	NDH+YFP	pABPURO	Constitutive expression	Puromycin
MIP-v5	29–13	MIP-V5	pT7-v5	Overexpression with c-term V5 tag	Puromycin

Inducible overexpression of MIP was performed by cloning the full-size gene into the pT7-V5 vector, for the addition of a C-terminal V5-tag to the open reading frame (ORF) [44]. *T*. *brucei* MIP ORF amplicon was obtained by PCR with forward 5´-GAAAAGCTTATGTTGCGGCGTGTCACC-3’ and reverse primers 5’- CCGGATCCCACCCATATGTCGATTTCAT-3’, flanked with restriction sites *Hind*III and *Bam*HI (underlined) for cloning into the expression vector. The final construct was digested with *NotI* for linearization prior to transfection. *In situ* tagged versions of beta-MPP were prepared in the alpha-MPP and MIP RNAi cell lines, using a PCR approach with long primers to produce a cassette that inserts the C-terminal V5 tag after the ORF, followed by a puromycin resistance gene [45]. The pPOTv4 vector was modified to bear a V5 tag and a puromycin resistance gene and was used as a template for PCR tagging.

To observe the processing activity of MIP, and compare it with that of alpha-MPP and beta-MPP, a construct composed of the N-terminal part containing the pre-sequence of a putative mitochondrial NADH-ubiquinone complex I subunit (NDH) (Tb927.11.13910) fused to YFP was prepared. The chimera was achieved by cloning the first 300 nucleotides at the 5’ of the YFP ORF, which lacked the ATG codon. For constitutive overexpression, the NDH-YFP gene fusion was cloned into the pABPURO vector [46] and transfected into the procyclic cell lines with inducible RNAi against alpha-MPP, beta-MPP, and MIP. All transfectants were selected via resistance to puromycin. The pre-sequences were analyzed for mitochondrial import signal in PSORTII and the MIP cleavage motif [42] using the *T*. *brucei* mitoproteome [41].

### Immunofluorescence assay

MIP was immunolocalized using its V5-tagged inducibly expressed version. Following an overnight induced overexpression triggered by the addition of 1 μg/ml of tetracycline to the medium, cells were incubated with MitoTracker Red for 20 min as previously described [47]. Subsequently, they were fixed with 4% (w/v) paraformaldehyde in phosphate buffered saline (PBS) and then permeabilized with 0.2% (v/v) Triton X-100 in PBS. Incubation with monoclonal anti-V5 antibody (Life Technologies) was performed for 1 hr in PBS mixed with 0.5% (w/v) gelatin in 1:1,000 dilution. The secondary antibody was anti-mouse Alexa Fluor 488 in 1:2,000 dilution (Life Technologies). DNA was stained with ProLong^®^Gold antifade reagent with 4',6-diamidine-2'-phenylindole dihydrochloride (DAPI) (Molecular Probes). The immunofluorescence assay was performed using a Zeiss microscope Axioplan 2, equipped with an Olympus DP73 digital camera.

### Western blot analysis

Expression of the target protein was compared between uninduced and induced RNAi cell cultures using cell lysates corresponding to 5 x 10^6^ cells per lane. Lysates were prepared in NuPAGE^®^ LDS sample buffer (Invitrogen), separated on Bolt 4–12% Bis-Tris polyacrylamide gels (Invitrogen) and transferred to an Amersham Hybond P PVDF membrane (GE Healthcare) for probing with the respective antibodies. Rat anti-alpha MPP, and rabbit anti-MIP were used at dilutions 1:1,000, 1:1,000 and 1:5,000, respectively. Polyclonal anti-enolase [48] and anti-trCOIV antibodies [49] were used at dilutions 1:50,000 and 1:10,000, respectively. Monoclonal anti-mtHsp70 was used at a dilution of 1:5000. Monoclonal anti-V5 (Invitrogen), polyclonal anti-GFP (Life Technologies) and monoclonal anti-tubulin antibodies (Sigma-Aldrich) were used at dilutions 1:2,500, 1:2,000 and 1:5000, respectively. Secondary antibodies were conjugated to horseradish peroxidase (Sigma-Aldrich), and the signal was visualized using Clarity Western ECL Blotting Substrate (Bio-Rad).

## Results

### Bioinformatic analysis of *T*. *brucei* MIP homolog

**Fig 1:**
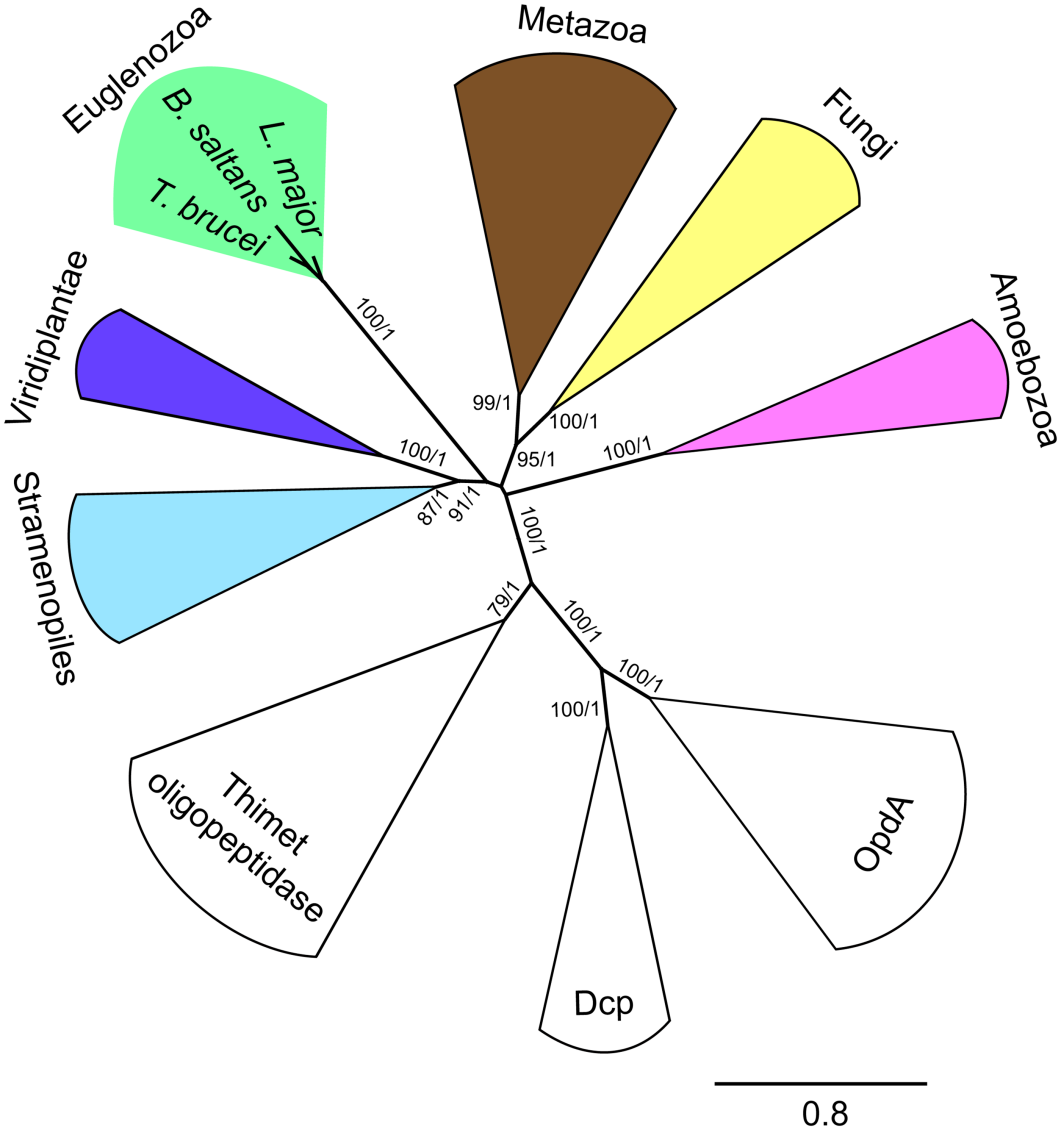
Phylogenetic analysis of *T*. *brucei* MIP and representative orthologs. Maximum-likelihood tree of MIP was constructed using 50 taxa and 340 sites. Bootstrap support and posterior probability values were calculated for each branch using PhyML and MrBayes, respectively. Close relatives to MIP, thimet oligopeptidase homologs, were used as outgroups. Only bootstrap and posterior probability values greater than 50% and 0.5, respectively, are shown. Chosen taxa and accession numbers are shown in S2 Table.

The gene encoding a putative MIP protein was annotated in the *T*. *brucei* genomic database (www.tritrypdb.org) under accession number (Tb927.10.9820). The protein sequence consists of 675 amino acid residues (76.7 kDa). MitoProt II [40] and PSORTII [38] predicted its mitochondrial localization with 88% and 70% probability, respectively. The putative MPP cleavage site was predicted at position 27 from the N-terminus. The zinc-binding site HEXXH is contained within conserved signature motif F-H-E-X-G-H-(X)_2_-H-(X)_12_-G-(X)_5_-D-(X)_2_-E-X-P-S-(X)_3_-E-X which is present across all MIPs and thimet oligopeptidase-like proteases (OpdA, Dcp, Thimet) [50]. This motif was found at positions 447–485 of the *T*. *brucei* homolog with only one change in position 479 where proline is replaced with phenylalanine. Phylogenetic analysis of MIP homologs representing all major eukaryotic supergroups reflected their relationships and is consistent with their monophyletic origin (Fig 1). Thimet peptidases, OpdA and Dcp were used as an outgroup to confirm that *T*. *brucei* MIP homolog belongs in MIP group and not in thimet oligopeptidase like proteases (Fig 1). Collectively, these predictions strongly indicate that the analyzed *T*. *brucei* gene encodes a genuine MIP.

### *T*. *brucei* MIP is an essential mitochondrial protein

**Fig 2:**
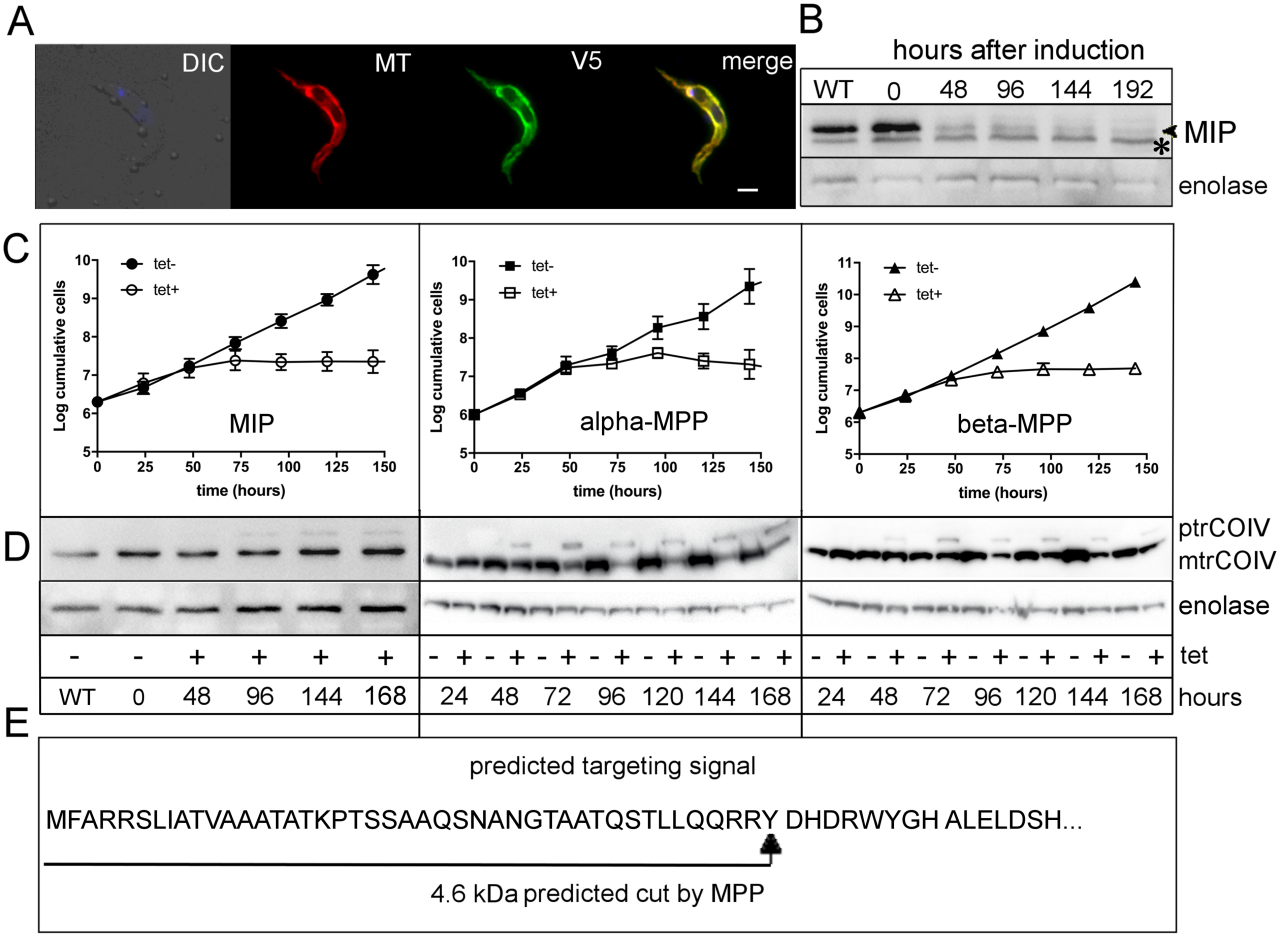
Subcellular localization and depletion phenotype of MIP. **A**) Immunofluorescence assay of MIP-V5 in procyclic stage *T*. *brucei*. Cells were incubated with MitoTracker (MT) (red), followed by immunodecoration with monoclonal anti-V5 antibody (V5) (green). DNA was stained using DAPI (blue). MIP was detected in the mitochondrion, as shown by the co-localization of the overexpressed protein with the MitoTracker signal (merge). *In situ* V5-tagged MIP showed identical localization results as the overexpressed protein (data not shown). **B**) Western blot analysis revealing the down-regulation of MIP by RNAi (indicated with a black arrowhead). The protein was efficiently depleted within 48 hrs of induction; * unspecific band. **C**) Growth curves of MIP, alpha-MPP and beta-MPP RNAi cell lines. Uninduced (black symbols) and RNAi-induced cell lines (white symbols). Three independent repeats were performed for each growth curve. **D**) Western blot analysis of trCOIV in cell lysates from RNAi cell lines for the respective peptidases, depicting accumulation of the precursor (ptrCOIV) and corresponding decrease of the mature protein (mtrCOIV). Enolase was used as a loading control. (+ tet) = RNAi-induced cells; (- tet) = uninduced cells. **E**) Schematic representation of the N-terminal sequence of trCOIV, displaying the predicted site for MPP cut. WT = wild-type cells; DIC = differential interference contrast; tet = tetracycline.

To confirm the predicted intracellular localization, the putative *T*. *brucei* MIP homolog was cloned into a pT7-v5 vector with the V5 tag attached to the 3’ end of the gene. Inducibly overexpressed MIP completely co-localized with the mitochondrial dye MitoTracker. The signal was evenly distributed throughout the mitochondrion (Fig 2A). Therefore, we conclude that MIP displays an exclusively mitochondrial localization in procyclic *T*. *brucei*.

A monitored depletion of MIP by RNAi by Western blot analysis showed an almost complete disappearance of the corresponding band after 48 hrs of induction (Fig 2B). As a consequence, the cells entered growth arrest (Fig 2C; left panel). Upon depletion of MIP, the trypanosomatid-specific subunit of cytochrome *c* oxidase (trCOIV) was visualized by Western blot analysis at different time points following RNAi induction. Accumulation of the trCOIV pre-protein was observed after 48 hrs of MIP RNAi induction (Fig 2D; left panel). The predicted N-terminal targeting sequence of trCOIV, calculated to be 4.6 kDa long, accumulated following MIP depletion and displayed a molecular weight as the one observed, when either alpha- or beta-MPP were RNAi-depleted (distinguishable in a 4–12% SDS-PAGE gel) (Fig 2D; central and right panels). This observation questions the presumption that trypanosomal MIP cleaves off an octapeptide. Furthermore, the substrates identified in trypanosomes differ from the ones found in yeast. At least this was the case of trCOIV, in which we were unable to find the MIP cleavage motif. Therefore to identify putative substrates of *T*. *brucei* MIP, we analyzed the mitoproteome by [41]. By using Gavel’s consensus patterns search in the PSORTII program to predict N-terminal targeting sequences with the MPP cleavage site, in the *T*. *brucei* mitoproteome composed of 403 proteins, 336 carried a recognizable targeting sequence. In the following analysis, proteins were filtered for the bulky hydrophobic amino acid motif F/L/I at position +1 from the MPP cleavage site and the G/T/S residue at position +4 to estimate MIP processing, which resulted in a set of 23 proteins likely processed by MIP (S2 Table). These proteins belong to a wide variety of pathways, mostly to RNA editing and processing, mitochondrial translation and transport across the mitochondrial membrane. One of the predicted substrates is NADH-ubiquinone oxidoreductase complex I subunit (Tb927.11.13910) (NDH), which we chose for further studies.

### YFP chimera with mitochondrial targeting signal

To assess processing by MIP, we designed a chimera protein with a long mitochondrial targeting signal, easily observable in Western blots. From the list of proteins predicted to contain the MPP/MIP cleavage sites, we selected a subunit of NADH-ubiquinone oxidoreductase complex I (Tb927.11.13910) that displays a predicted targeting sequence of 85 amino acids. Its N-terminal sequence was fused to YFP to determine how is the chimera processed in each of the three RNAi cell lines (alpha-MPP, beta-MPP, and MIP). The chimera construct was stably transfected and the resulting cell line was assessed by immunofluorescence microscopy to ensure that the chimeric protein was successfully translocated into the mitochondrion.

**Fig 3:**
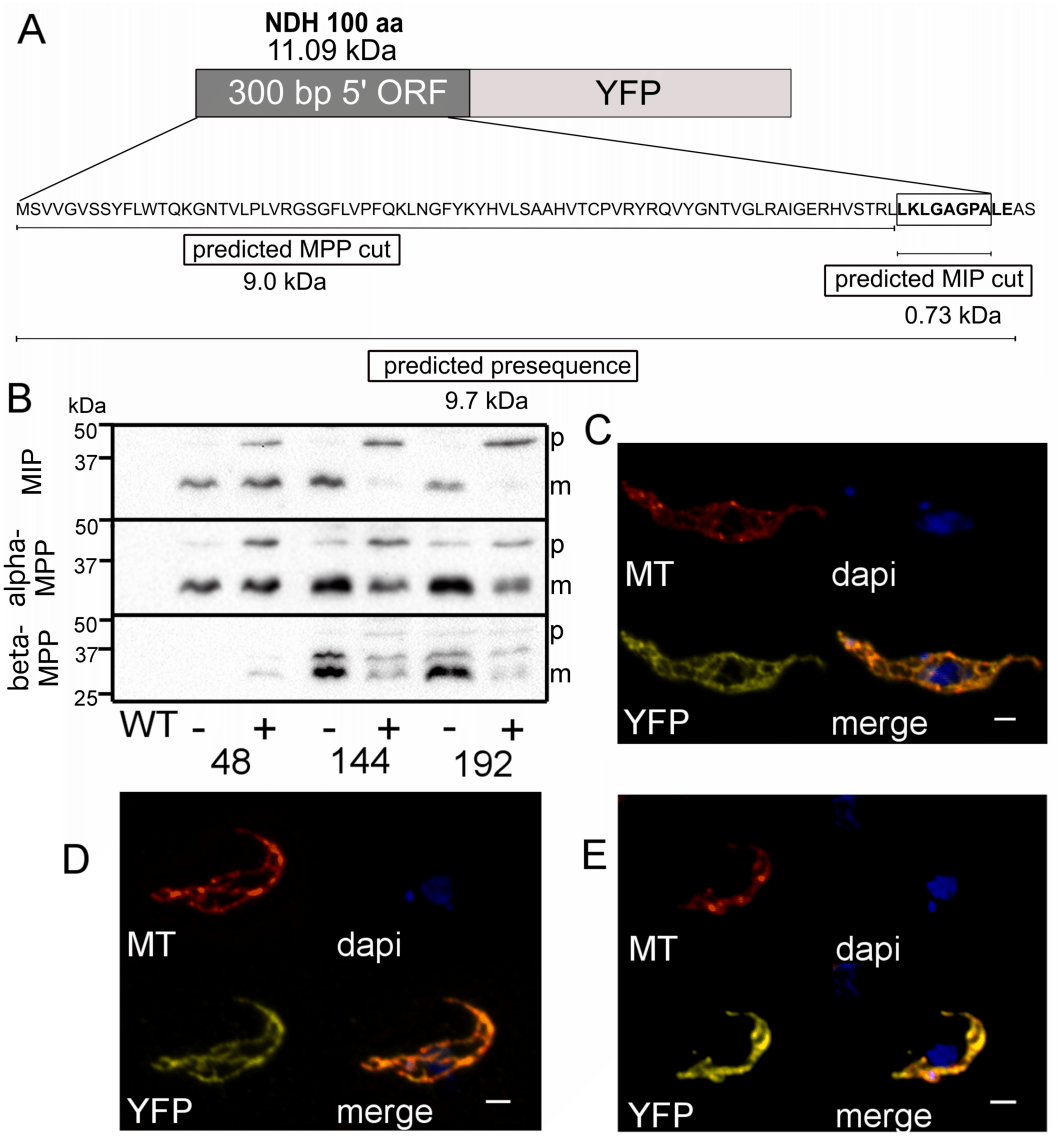
Processing of a YFP-chimera in the background of the mitochondrial processing peptidases. Mitochondrial localization of the NDH-YFP chimera in trypanosomes RNAi-ablated for MIP. **A**) Schematic representation of the NDH-YFP chimera; the construct is composed of a stretch of 100 N-terminal amino acids from a putative subunit of complex I. The predicted sites for MPP and MIP cleavages are shown. **B**) Western blot analysis of the NDH-YFP protein in cells with inducible RNAi against the indicated peptidase. (+ tet), RNAi-induced cells; (- tet), uninduced cells; p, NDH-YFP precursor; m, mature NDH-YFP. Detection of the chimeric protein was perfomed using a polyclonal anti-GFP antibody. Subcellular distribution of this chimera was identical in RNAi cell lines of MIP (**C**), alpha-MPP (**D**) and beta-MPP (**E**). YFP signal is shown in yellow; MitoTracker (MT) is shown in red; DAPI stains DNA; merge shows all dyes at once. Scale bar is 1 μm.

A schematic representation of the size and sequence of the N-terminus that should undergo processing in the YFP chimera is shown in Fig 3. The YFP signal was expressed in all cells and indeed displayed mitochondrial localization (Fig 3). With either of the MPP subunits depleted, the predicted precursor of the NDH-YFP chimera, estimated to be approximately 10 kDa larger than the mature protein, accumulated. In the MIP RNAi cell line, the expected NDH-YFP precursor should be approximately 1 kDa larger than the mature protein (Fig 3A). However, in both the alpha-MPP and MIP RNAi cell lines, the accumulated precursor was exactly of the same size, about 10 kDa larger than the mature protein. When beta-MPP was down-regulated, two putative precursors accumulated; the first one likely corresponds to the precursor observed in the alpha-MPP RNAi cell line, whereas the second smaller one is reminiscent of the intermediate form expected in the MIP-depleted trypanosomes (Fig 3B).

### MIP downregulation affects MPP expression

**Fig 4:**
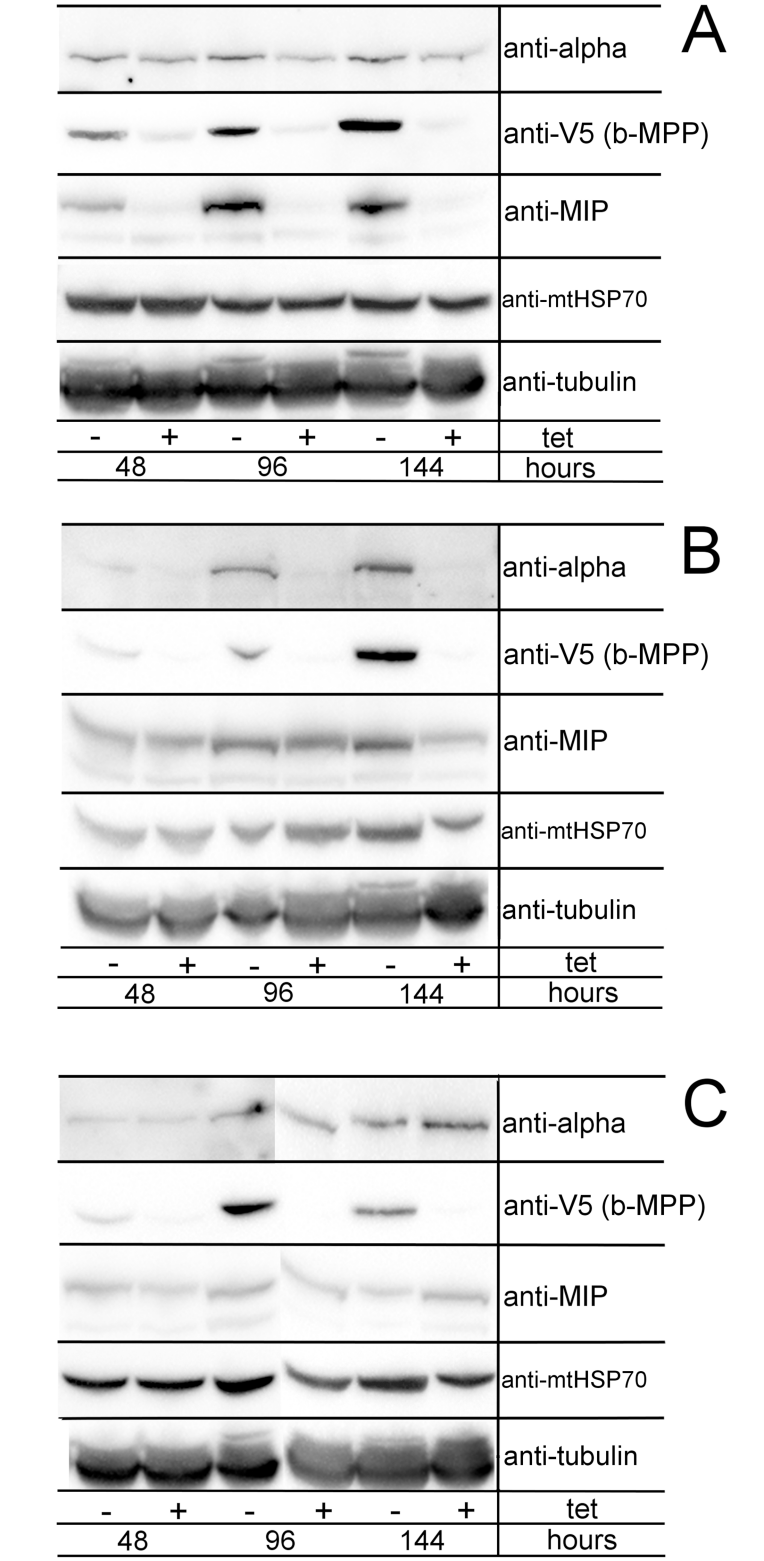
Assessment of co-expression of MIP, alpha-MPP, and beta-MPP in an RNAi background of each of the three peptidases. Beta-MPP was V5-tagged *in situ* in all three cell lines to monitor its expression by Western blot. 5 x 10^6^ cells/well were prepared in lysates for Western blot analysis. Samples were taken after 2, 4 and 6 days of RNAi induction. The levels of alpha-MPP, V5-tagged beta-MPP, MIP, and mtHSP70 were analyzed in all three cell lines. Tubulin was used as a loading control. **A**) MIP RNAi cell line. **B**) Alpha-MPP RNAi cell line. **C**) Beta-MPP RNAi cell line; (+ tet) = RNAi-induced cells; (- tet) = uninduced cells.

To determine whether down-regulation of MIP had any effect on the expression of the canonical MPP, we have tagged beta-MPP with V5 *in situ*, in the alpha-MPP, beta-MPP and MIP RNAi cell lines. When MIP was depleted via RNAi, beta-MPP was also down-regulated (Fig 4A). In the alpha-MPP RNAi cell line, depletion of the target protein lead to a parallel down-regulation of V5-tagged beta-MPP and to a lesser extent also of MIP (Fig 4B). In a similar fashion, downregulation of alpha-MPP was observed when beta-MPP was downregulated. However, RNAi was not accompanied by drastic changes in MIP levels (Fig 4C).

## Discussion

The processing of hundreds of nuclear-encoded proteins that are imported into the mitochondrion is a universal mechanism for eukaryotes [13,51,52]. The model protist *T*. *brucei* is no exception, as the RNAi-mediated down-regulation of the canonical MPP resulted in a growth arrest phenotype [31]. Although some protists were shown to harbor an unusual MPP formed by a single-subunit [53], trypanosomes possess a canonical MPP enzyme functioning as a heterodimer of alpha and beta subunits [32], similar to the well-described homologs in yeast and mammals [14,54]. AtOct1, the *A*. *thaliana* homolog of MIP, initially associated with the thylakoid membrane, has also been localized to the mitochondrion [28,55,56]. Being no exception, the *T*. *brucei* MIP protein displays an exclusively mitochondrial localization.

Here we show that MIP is an essential mitochondrial peptidase. This contrasts with the results obtained for yeast Oct1 depletion, where the downregulation impaired growth in non-fermentable carbon sources, but did not completely abolish cell viability [26]. Since fermentative metabolism allows yeast to survive in the presence of sugars, the function of Oct1 in this organism has been linked to the processing of nuclear-encoded subunits of mitochondrial respiratory complexes, hence the growth defect in non-fermentable carbon sources [23]. On the other hand, due to its aerobic fermentative metabolism, the procyclic *T*. *brucei* makes use of its oxidative phosphorylation machinery for energy production when glucose is not available [57,58]. However, the growth medium used for *in vitro* cultivation of this flagellate contains glucose in concentrations high enough to support ATP production via glycolysis [59]. Therefore, procyclic trypanosomes analyzed herein mimic fermentative metabolism but in parallel use oxygen as a final electron acceptor. Based on the predicted substrates of *T*. *brucei* MIP, it is obvious that the set of putative MIP targets is different from those identified in *S*. *cerevisiae* [23,60]. However, in both organisms, MIP cleaves proteins that participate in core mitochondrial pathways. Fourteen confirmed Oct1 substrates in *S*. *cerevisiae* belong to DNA binding and mitoribosomal proteins and subunits of the respiratory chain, while 23 putative substrates in *T*. *brucei* identified herein also include mitoribosomal subunits, but most belong to protein complexes involved in RNA editing and processing.

Out of the subset of yeast‘s Oct1 substrates, COXIV was found to accumulate a precursor when the protease was downregulated [23]. Though in procyclic trypanosomes trCOIV is not a homologue of COXIV in yeast, it is an essential subunit of cytochrome *c* oxidase for which a specific antibody is available [49]. In cells depleted for MIP, a processing defect of trCOIV occurred, reflected by the accumulation of the corresponding precursor, though trCOIV was not found to be a predicted substrate of MIP through sequence analysis. However, the observed precursor cannot be reconciled with MIP trimming off just the canonical octapeptide, as would be assumed based on what is known about this peptidase in yeast. The pre-processed trCOIV accumulated in the form of an approximately 40 kDa-long precursor, yet exactly the same-size precursor accumulated also in cells ablated for either alpha- and/or beta-MPP.

Another important event triggered by the depletion of MIP is the reduced expression of beta-MPP. Alternatively, the amount of MIP in cells depleted for either alpha-MPP or beta-MPP drops, but the down-regulation is not as severe. As a typical mitochondrial protein, MIP requires processing by MPP prior to folding and entry into an enzymatically active conformation. However, the opposite does not seem to be the case from the point of view of octapeptide sequences, since neither alpha-MPP nor beta-MPP display the octapeptide sequence [61]. This does not necessarily reflect that the *T*. *brucei* MIP requires the conserved octapeptide to trim all of its substrates, a provision further supported by the fact that the *A*. *thaliana* MIP was shown not to recognize such an element. In fact, the octapeptide seems to be absent from the pool of mitochondrial proteins in this model plant, making its MIP an example of a conserved, yet also adaptable peptidase for achieving stable N-terminal proteolytic processing of imported proteins [28]. Hence, we speculate that the growth phenotype observed in our study is most likely caused by the depletion of several essential mitochondrial proteins that require processing by MIP, and also by the subsequent down-regulation of beta-MPP, which in turn affects the processing of a much wider array of mitochondrial substrates. Though it cannot be completely ruled out that *in situ* tagging of beta-MPP may have affected its activity and/or capability for protein-protein interaction, the differential relationships between beta-MPP and alpha-MPP, and beta-MPP and MIP, denote that the observed effect is specific for beta and MIP.

The expression of the YFP-chimera demonstrated that the impairment of MPP activity in cells depleted for MIP is not confined to the processing of trCOIV. Expression of the NDH-YFP chimera in the alpha-MPP RNAi background resulted in the accumulation of a precursor by approximately 10 kDa bigger than the mature protein. Since the precursor should be removed by MPP in trypanosomes depleted for MIP, the predicted precursor should be much smaller than the one observed. However, since depletion of MIP causes downregulation of beta-MPP, the precursor was not processed.

In the beta-MPP RNAi cells, the NDH-YFP chimera displays a pre-processed intermediate significantly different from that observed in trypanosomes depleted for either MIP or alpha-MPP. In the absence of beta-MPP, NDH-YFP precursors of two sizes are detected: the larger precursor is similar to the one observed in the alpha-MPP and MIP RNAi cell lines, while the smaller precursor is slightly bigger than the mature protein. The presence of this band may represent a mechanism by which the cell copes with the absence of one component of the MPP heterodimer. At the same time, this result led us to postulate that these proteases may not necessarily act in the same way as they do in yeast and that their concerted activity may vary from substrate to substrate.

Unfortunately, numerous attempts to dissect the processing using an *in vitro* activity assay failed. We hypothesise that TbMIP may function synergically with MPP, probably stabilizing or in association with the mitochondrial MPP.

## Supporting information

S1 Table Accession numbers of MIP homologs, thimet, OpdA and Dcp oligopeptidases used for the phylogenetic reconstruction in Fig 1. (XLSX)

S2 Table Putative MIP substrates present in the *T*. *brucei* mitochondrion. The sequences of mitochondrial proteins were obtained from (40) and assessed for the presence of the N-terminal mitochondrial targeting sequence with MPP and MIP cleavage site motives. MIP octapeptide target sequence was identified based on the amino acid residue at the position +1 (F/L/I) and +4 (T/S/G), relative to the MPP cleavage site.
(XLS)
